# Effect of clinical decision rules, patient cost and malpractice information on clinician brain CT image ordering: a randomized controlled trial

**DOI:** 10.1186/s12911-018-0602-1

**Published:** 2018-03-12

**Authors:** Ronald W. Gimbel, Ronald G. Pirrallo, Steven C. Lowe, David W. Wright, Lu Zhang, Min-Jae Woo, Paul Fontelo, Fang Liu, Zachary Connor

**Affiliations:** 1 Department of Public Health Sciences, Clemson University, 501 Edwards Hall, Clemson, SC 29634-0745 USA; 2 Department of Emergency Medicine, Greenville Health System, Greenville, SC USA; 3 Department of Radiology, Greenville Health System, Greenville, SC USA; 4 Department of Emergency Medicine, Emory University, Atlanta, GA USA; 5 Lister Hill National Center for Biomedical Communication, National Library of Medicine, Bethesda, MD USA

**Keywords:** Clinical decision making, CT brain, Mild head trauma, Patient cost information, Simulation research, Malpractice information, Canadian CT head rule, Emergency department clinicians, Evidence-based medicine

## Abstract

**Background:**

The frequency of head computed tomography (CT) imaging for mild head trauma patients has raised safety and cost concerns. Validated clinical decision rules exist in the published literature and on-line sources to guide medical image ordering but are often not used by emergency department (ED) clinicians. Using simulation, we explored whether the presentation of a clinical decision rule (i.e. Canadian CT Head Rule - CCHR), findings from malpractice cases related to clinicians not ordering CT imaging in mild head trauma cases, and estimated patient out-of-pocket cost might influence clinician brain CT ordering. Understanding what type and how information may influence clinical decision making in the ordering advanced medical imaging is important in shaping the optimal design and implementation of related clinical decision support systems.

**Methods:**

Multi-center, double-blinded simulation-based randomized controlled trial. Following standardized clinical vignette presentation, clinicians made an initial imaging decision for the patient. This was followed by additional information on decision support rules, malpractice outcome review, and patient cost; each with opportunity to modify their initial order. The malpractice and cost information differed by assigned group to test the any temporal relationship. The simulation closed with a second vignette and an imaging decision.

**Results:**

One hundred sixteen of the 167 participants (66.9%) initially ordered a brain CT scan. After CCHR presentation, the number of clinicians ordering a CT dropped to 76 (45.8%), representing a 21.1% reduction in CT ordering (*P* = 0.002). This reduction in CT ordering was maintained, in comparison to initial imaging orders, when presented with malpractice review information (*p* = 0.002) and patient cost information (p = 0.002). About 57% of clinicians changed their order during study, while 43% never modified their imaging order.

**Conclusion:**

This study suggests that ED clinician brain CT imaging decisions may be influenced by clinical decision support rules, patient out-of-pocket cost information and findings from malpractice case review.

**Trial registration:**

NCT03449862, February 27, 2018, Retrospectively registered.

**Electronic supplementary material:**

The online version of this article (10.1186/s12911-018-0602-1) contains supplementary material, which is available to authorized users.

## Background

Minor head trauma is a common condition treated by emergency department (ED) clinicians [1, 2]. Over one million computed tomography (CT) scans are conducted annually in the United States for these patients, with less than 10% demonstrating findings that change medical management [3–5]. The need for brain CT in patients with minor head trauma has been thrust into the national spot light because of concerns about the long-term danger of low-dose radiation exposure and the desire to decrease unnecessary health care costs [4, 6].

There are numerous reasons why clinicians may order advanced medical imaging for patients despite clinical evidence suggesting otherwise. These issues include, but are not limited to, unfamiliarity or mistrust of clinical decision rules [5, 7] and estimation of radiation burden [8–11], fear of malpractice lawsuits [12–14], and lack of knowledge of the cost of medical imaging.

With respect to minor head trauma, several validated evidence-based clinical decision rules have been published to help guide clinicians in ordering brain CTs [3, 5, 15]. While these rules differ with respect to sensitivity and specificity, they provide a medically and legally justified pathway to support decision making. Despite this, evidence suggests that many clinicians do not follow clinical decision rules unless reinforced by practice policy and/or integrated into clinical work flow [12, 16].

It is well documented in the literature that “clinician fear of a malpractice suit” exists, and influences clinical decision making [3, 12, 14, 17]. Fear of a lawsuit has led to increased CT ordering, despite the existence and validation of clinical decision rules [16, 18].

Finally, substantial literature reveals that clinicians are unfamiliar with cost implications of testing to patients, payers, and health systems [19–21]. However, evidence suggests that clinician awareness of testing cost may influence their decision making, especially toward less-costly testing options [21–23]. One abstract even shows that insured medical patients are more likely to receive a brain CT in cases of minor head injuries than patients without health insurance [16].

This study expands our previous research on how information influences clinician decision making in medical image ordering for adult and pediatric patients in the primary care environment [23, 24]. In our earlier work, we incorporated simulation-based methodology to explore how clinician medical image ordering behavior might be influenced by the introduction of clinical decision rules, estimated radiation exposure information, and estimated cost. We analyzed the temporal effect of information presentation to clinicians and the relationship between clinician demographics and medical image ordering behavior.

## Goals of the investigation

In our current study, we seek to explore whether presentation of the Canadian CT Head Rule (CCHR), findings from a medical-legal review of malpractice cases related to lack of CT ordering in head trauma victims, and estimated patient out-of-pocket cost for CT imaging might influence clinician ordering in response to clinical vignettes in the simulated emergency department environment. Our goal was to further inform practicing emergency department clinicians and the medical informatics community as they collaborate to build clinical decision support systems that aid clinical decision making.

## Methods

### Study design and setting

This was a multi-center, double-blinded, with balanced ([1:1]) randomization, parallel-group study conducted in the United States with the Departments of Emergency Medicine, Greenville Health System (Greenville, SC) and Emory Healthcare (Atlanta, GA). The study was approved by the Institutional Review Boards of both health systems. There were no changes to the methods after the simulation trial had commenced. As this was a web-accessible simulation study, participants could participate in the study anywhere connectivity allowed. Participants could access the simulation study via a computer or portable device (e.g. tablet) that was internet-accessible.

### Selection of participants

Study participation was limited to clinicians, with image ordering capability for patients, and employed in the emergency department of one of the two health systems. Clinicians included attending physicians, resident physicians, physician assistants and nurse practitioners Randomization occurred when the clinicians indicated their occupational profession (see above). Specifically, an electronic balancer allocated each participant (by occupational profession) to one of two groups. The two groups were the LEGAL-COST group or the COST-LEGAL group, both described below, which differed in the temporal order in which they were presented information in the study. The study was double-blinded in that neither the investigators nor the participants were aware of the order in which legal and cost information would be presented. For clarity, both groups received all information which differs from a traditional interventional trial with intervention and control groups.

### Recruitment

An electronic email invitation from each department’s academic leadership was sent to all ED clinicians and EM residents. Reminder emails were sent to all at approximately 14 days and 21 days after the initial invitation. Leadership was blinded to participation, assuring voluntary participation without fear of repercussion.

### Interventions

Our intervention consisted of two clinical vignettes, follow-up decision screens and collection of demographic data (Additional file 1). Both clinical vignettes were jointly developed by the emergency medicine and radiology clinician study authors. The cases were designed to provide sufficient information for the clinician to make a determination regarding need for CT imaging. Both cases were designed to fall below the threshold, as outlined in the CCHR, for requiring CT imaging. To further unify the cases both patients had known normal renal function and no allergy to contrast.

Following an electronic-based informed consent process, participants were presented with clinical vignette #1 which described a 58-year-old female (simulation patient) who presents to the Emergency Department after falling on ice hitting her head on the sidewalk (Additional file 1). Following the case presentation, the clinicians were prompted to make a medical image ordering decision for this patient among three options: CT brain (without and with contrast), CT (without contrast), or no imaging.

After their initial imaging decision, the clinicians were presented with the brain CT ordering criteria based on the CCHR [1, 15]. We included a hyperlink to three manuscripts (2 abstracts, 1 full-text) supporting the criteria for clinicians who desired to review further material [15, 25, 26]. The clinicians were provided a first opportunity to modify their initial imaging order.

After their opportunity to modify based on the CCHR, the next topic presented was estimated out of pocket costs regarding the expense of the ED visit with and without imaging. The costs were based on actual local ED charges with the average patient out-of-pocket expense after insurance for a brain CT identified as $843. This was derived from actual data (year 2015) calculated from a Level 1 Trauma Center in the Southeast United States. Following presentation of this information, clinicians were provided a second opportunity to modify their initial imaging order.

The third topic presented was a collection of findings from a malpractice case law review (years 1972–2014) covering situations where the clinician did not order a brain CT for a minor head trauma. Participants were provided with an additional hyperlink to the original published article for review [3]. The malpractice law review was included as an evidence source that addresses “clinician fear of malpractice lawsuit if not ordering a brain CT for minor head trauma”. Following presentation of this information, clinicians were provided a third and final the opportunity to modify their initial imaging order.

After all of the information was presented, constituting the breadth of the intervention, vignette #2 was presented to assess how clinicians might apply their new knowledge for similar scenarios moving forward. Vignette #2 describes a 62- year old female (simulation patient) who had a witnessed slip and fall at home (Additional file 1). Following this case presentation, the clinicians were given a single opportunity to make a medical image ordering decision among three options: CT brain (without and with contrast), CT (without contrast), or no imaging. Thus, a total of 5 clinical decisions were recorded for each participant based on 2 cases.

Demographic and general survey data were gathered from participants. Demographic data included age, gender, role (i.e. practicing clinician, trainee), and years of clinical practice. Two exploratory questions on the participant’s economic attitudes required responses presented on a Likert-like 1–7 scale. The first was “Making better use of my resources makes me feel good”; participants were asked to agree or disagree with the statement. The second was “I believe in being careful in how I spend my money”; participants were asked to agree or disagree with the statement. Both of these questions were derived from the consumer-oriented literature where the focus was on measurement of consumer frugality [27].

Our study concluded with an option for participants to earn a no-cost one Category 1 AMA physician continuing medical education (CME) credit. Participants were redirected from our study website to the Continuing Medical Education Office of the Greenville Health System. There participants reviewed summary material from our study, were provided the opportunity to review full-text reference papers, then complete a post-education survey assessing comprehension to receive CME credit.

### Methods and measurements

**Fig. 1:**
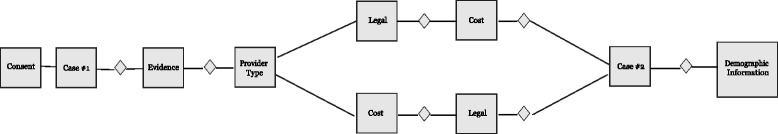
Study Flow. Note: Diamonds denote clinician medical imaging decision point

As presented in Fig. 1, and described above, participants made image ordering decisions at five points in the study. The decisions were made by the participants within the simulation study and recorded in our server-based analysis database in descriptive form (e.g. no imaging, brain CT without contrast) in Microsoft Excel® format. A copy of the database was distributed to researchers at Clemson University where the descriptive data were properly coded for analysis by two researchers (MW, RG). The coded file was then imported into SAS, v.9.4 (Cary, NC).

Following the second image ordering decision point the participants were asked how they signed orders and prescriptions (i.e. as a physician, nurse practitioner, physician assistant, or other). Based on the response, participants were stratified by clinician type and balanced randomized [1:1] to one of two parallel arms (i.e. LEGAL-COST group or the COST-LEGAL group). Stratification was used to ensure the clinician types were equally distributed and thereby ensuring the two arms were homogeneous. The difference between the two arms was the temporal presentation of supplemental decision information. The LEGAL-COST group was presented information on malpractice case law then patient out-of-pocket cost information. The COST-LEGAL group was presented information on patient out-of-pocket cost information then malpractice case law information. The participant study flow is shown in Fig. 1.

### Outcomes

The primary outcome measure for the study was the physicians’ selection of imaging tests after receiving CCHR information, malpractice case review information, and patient out-of-pocket expense information. We also measure the physicians’ selection of imaging tests immediately following clinical vignette #1 and after presentation of clinical vignette #2.

### Analysis

Data were recorded in our intervention server, housed at the National Institutes of Health, in a Microsoft Excel spreadsheet. The data were downloaded and analyzed using SAS, ver. 9.4 (Cary, NC). In our study, because clinicians’ ordering image vs. not ordering image was the main comparison of interest, CT brain modalities (with or without contrast) were grouped together and compared with the no imaging order group. To compare the clinicians’ change in image ordering, McNemar test was employed and a multiple comparison adjustment was performed using Bonferroni correction. For the comparison of the demographic or other professional characteristics among different clinician groups, Chi-square test was employed to compare the proportions of categorical variables; if more than 20% of the cells had sample size less than 5, Fisher Exact test was applied instead. Analysis of Variance (ANOVA) test was used to compare the mean (standard deviation) of continuous variables.

A sample size of 155 will achieve 80% power to detect a difference in the proportion of selecting CT imaging if 30% of clinicians choose CT order in the absence of evidence and change to no imaging order when the evidence is presented and 15% of clinicians choose no imaging order in the absence of evidence and changed to CT order when the evidence is presented.

## Results

### Characteristics of study subjects

Participants in the study included 150 emergency medicine physicians, 12 nurse practitioners, and 5 physician assistants allocated to one of 2 groups; the LEGAL-COST group (*n* = 82) and the COST-LEGAL group (*n* = 85). There was one clinician missing the initial imaging order and another five clinicians missing the 3rd order. These participants were included in the study sample and analyzed. All other data items were complete.

Approximately 90% of the participants were practicing clinicians with 10% being trainees; balanced among the 2 groups. Gender was balanced with slightly more males than females participating in both groups. Approximately two thirds of participants had > 5 years of clinical practice experience; included in the group were the > 40% with > 10 years of clinical practice. Just over half of the participants were ≤40 years of age (Table 1). There were no significant differences between the 2 groups with respect to demographics (Table 1) or initial image ordering decisions to clinical vignette #1 (Table 2).

**Table 1:** Participant demographics

	LEGAL-COST group (*n* = 82)	COST-LEGAL group (*n* = 85)	*P*-value*	Both groups (*n* = 167)
Clinician type			0.92	
Physician	73 (89.0%)	77 (90.6%)		150 (89.8%)
Nurse Pract.	6 (7.3%)	6 (7.1%)		12 (7.2%)
Physician Assist.	3 (3.7%)	2 (2.4%)		5 (3.0%)
Role			0.74	
Practicing clinician	73 (89.0%)	77 (90.6%)		150 (89.8%)
Trainee	9 (11.0%)	8 (9.4%)		17 (10.2%)
Years of Practice			0.31	
Less than 5 years	32 (39.0%)	24 (28.2%)		56 (33.5%)
5–10 years	17 (20.7%)	23 (27.1%)		40 (24.0%)
More than 10 years	33 (40.3%)	38 (44.7%)		71 (42.5%)
Gender			0.23	
Male	49 (59.8%)	43 (50.6%)		92 (55.1%)
Female	33 (40.2%)	42 (49.4%)		75 (44.9%)
Age (year)			0.40	
< 30	6 (7.3%)	10 (11.8%)		16 (9.6%)
31–40	40 (48.8%)	34 (40.0%)		74 (44.3%)
41–50	14 (17.1%)	21 (24.7%)		35 (20.9%)
51+	22 (26.8%)	20 (23.5%)		42 (25.2%)

*P-value was calculated using Chi-square test except for the comparison of clinician type, *P*-value was calculated using Fisher exact test (more than 20% of cells with sample size less than 5)

Notes: LEGAL-COST indicates exposure to legal then cost information; COST-LEGAL indicates exposure to cost then legal information

**Table 2:** Proportion of computed tomography ordering in clinician medical image order decisions

		Case #1				Case #2
		Initial choice (Initial order)	CCHR (2nd order)	Legal (3rd order for Legal-Cost group; or 4th order for Cost-Legal group)	Cost (4^th^order for Legal-Cost group; or 3rd order for Cost-Legal group)	Final choice (5th order)
Both groups	n (%)	116 (66.9%)	76 (45.8%)	85 (52.5%)	80 (47.9%)	109 (65.7%)
(*n* = 167)	*P*-value^*^		Initial vs. CCHR: 0.002	Initial vs. Legal: 0.002	Initial vs. Cost: 0.002	Initial vs. Final: 1.00
LEGAL-COST^a^	n (%)	56 (68.3%)	36 (43.9%)	38 (49.4%)	39 (47.6%)	50 (61.0%)
(*n* = 82)	*P*-value^*^		Initial vs. CCHR: 0.003	Initial vs. Legal: 0.05 CCHR vs. Legal: 0.8	Initial vs. Cost: 0.01 Legal vs. Cost: 1.00	Initial vs. Final: 1.00
COST-LEGAL^b^	n (%)	60 (71.4%)	40 (47.6%)	47 (55.3%)	41 (48.2%)	59 (70.2%)
(*n* = 85)	*P*-value^*^		Initial vs. CCHR: 0.002	Initial vs. Legal: 0.05 Cost vs. Legal: 1.00	Initial vs. Cost: 0.002 CCHR vs. Cost: 1.00	Initial vs. Final: 1.00

*Abbreviations*: *CCHR* Canadian CT Head Rule

*P-values were calculated using McNemar test and a multiple comparison adjustment was performed using Bonferroni correction

^a^LEGAL-COST indicates exposure to legal then cost information

^b^COST-LEGAL indicates exposure to cost then legal information

### Main results

Clinician image ordering decisions are presented in Table 2. For clinical vignette #1, 116 of the 167 participants (66.9%) initially order a CT image. After presentation of the CCHR, with option to access abstracts or full-text manuscript supporting the rule, the number of clinicians ordering a CT image dropped to 76 (45.8%), which represents a 21.1% statistically significant (*P* = 0.002) reduction in CT ordering in favor of no medical imaging. It is noteworthy that only 7.8% (*n* = 13) of the 167 participants accessed either ≥1 abstract (*n* = 6) or full-text manuscript (*n* = 7) indexed to the decision screen prior to making their imaging decision.

Following the CCHR-related imaging decisions, clinicians were presented with either LEGAL (LEGAL-COST group) or COST (COST-LEGAL group) information. In the LEGAL-COST group, after receiving information about malpractice judgements against clinicians who did not order a CT of the brain in mild trauma cases, the number of clinicians ordering a CT image was 38 (49.4%) and the difference was significant (*P* = 0.05) when compared to their initial order, and but not significant when compared to their previous image order that followed presentation of the CCHR. After presentation of patient out-of-pocket expense information, the number of clinicians ordering a CT was 39 (47.6%) where the difference was significant (*P* = 0.01) when compared with their initial order but not significant when compared to their previous image order that followed presentation of malpractice judgement information.

In the COST-LEGAL group, after presentation of patient out-of-pocket expense information, the number of clinicians ordering a CT image was 41 (48.2%) and the difference was significant (*P* = 0.002) when compared to their initial order, but not significant when compared to their previous image order that followed presentation of the CCHR. After receiving information about malpractice judgements, the number of clinicians ordering a CT was 47 (55.3%) and the difference was significant (*P* = 0.05) when compared with their initial order but not significant when compared to their previous image order that followed presentation of patient out-of-pocket expense information.

When comparing the clinician’s initial decision regarding medical imaging ordering in response to clinical vignette #1 to their decision in response to clinical vignette #2, the differences were not significant. It is noteworthy that the clinical scenarios in both vignette’s, albeit not precisely the same case, were within the criteria for no medical imaging necessary when applied to the CCHR. In clinical vignette #2, approximately two thirds of clinicians ordered a CT for their patients which is consistent with their response to clinical vignette #1 (Table 2).

Comparing the initial medical imaging decision and the three subsequent imaging decision options for clinical vignette #1, 49 (30.4%) of clinicians always ordered a CT image, 20 (12.4%) of clinicians never ordered a CT image, and 98 (57.2%) changed their CT image order at least once (Table 3). Of 36 participants who changed their imaging order more than once, 27 (16.2%) changed their order at least twice, 6 (3.6%) changed their order at least three times, and 3 (1.8%) modified their imaging order four times (data not shown in the table).

**Table 3:** Clinician demographics and imaging decisions (by CT ordering behavior group)

	Clinicians who always ordered CT (*n* = 49)	Clinicians who never ordered CT (*n* = 20)	Clinicians who changed CT order (*n* = 98)	*P*-value^a^	Total (*n* = 167)
Provider type, %				0.70	
Physician	89.8	95.0	88.8		89.8
NP/PA	10.2	5.0	11.2		10.2
Gender, %				0.75	
Male	59.2	50.0	54.1		55.1
Female	40.8	50.0	45.9		44.9
Years of clinical practice, %				0.09	
< 5	26.5	40.0	35.7		33.5
5–10	18.4	40.0	23.5		24.0
> 10	55.1	20.0	40.8		42.5
Resource question, mean (SD)	6.00 (1.13)	6.45 (0.69)	6.17 (0.95)	0.22	6.16 (0.98)
Money question, mean (SD)	5.73 (1.18)	6.30 (0.73)	6.00 (1.06)	0.11	5.96 (1.08)

^a^Chi-square test was employed to compare the proportion of categorical variable; Analysis of Variance (ANOVA) test was employed to compare the means of continuous variable

Notes: CT, computed tomography; NP, nurse practitioner; PA, physician assistant; SD, standard deviation; mean response to resource question and money question based on 1–7 Likert-like scaled response

The majority of clinicians who always ordered CT imaging had accumulated > 10 years in clinical practice and scored lower on both the use of resource and care with money questions as compared with clinicians never ordering CT or those that changed their imaging order, but the difference did not achieve statistical significance (Table 3). In contrast, the majority who never ordered a CT image had accumulated < 10 years in clinical practice and scored higher on both the use of resource and care with money questions as compared to clinicians who always ordered a CT image or those that changed their imaging order (Table 3).

When aggregating data from both groups comparing CT image ordering and response to the “use of resource question”, those participants who indicated a 7 score (strongly agree) on the “use of resource question” did not have a significantly different CT ordering behavior from those with a question score of 1–6 for clinical vignette #1. However, this shifted in clinical vignette #2 where the two groups were significantly different (*p* = 0.02); those scoring a 7 were more likely not to order a CT image while those scoring 1–6 were more likely to order CT image (Table 4). When aggregating data from both groups comparing CT image ordering and response to the “being careful in spending money” question, there were no statistical differences recognized (Table 4).

**Table 4:** Comparison of clinician response to attitudinal survey questions on resource utilization and spending money to CT ordering behavior

	Case #1 (initial order)			Case #2 (5^th^order)		
Survey question	CT	No image	*P*-value*	CT	No image	*P*-value*
Better use of my resources makes me feel good						
strongly agree (7)	51 (44.4%)	23 (46.0%)	0.84	42 (38.5%)	33 (57.9%)	0.02
< strongly agree (1–6)	64 (55.7%)	27 (54.0%)		67 (61.5%)	24 (42.1%)	
I believe in being careful in how I spend my money						
strongly agree (7)	40 (34.78%)	24 (48.0%) 26	0.11	38 (34.9%)	26 (45.6%)	0.18
< strongly agree (1–6)	75 (65.22%)	(52.0%)		71 (65.1%)	31 (54.4%)	

*P-value was calculated from Chi-square test

### Limitations

Our study was based in a simulation environment. The sounds, interruptions, clinical pressures, and triage triggers of emergency department workflow were absent. Furthermore, participants were not responsible for following policies or other considerations in the clinical scenarios. It is possible that the clinicians may have manipulated their medical imaging decisions in search of the “right answer” and did not truly consider the implication of new information in their clinical care. The clinicians may not have conceptualized that the decision screens and variables (i.e. evidence, cost, legal) were fully applicable in clinical scenario #2 in the same manner as scenario #1. This study includes participants from 2 southern US states that have different state tort and liability malpractice reforms and environments that may not be applicable to other US practice locations.

## Discussion

Evidence from our simulation-based study suggests that ED clinician decision making may be influenced by clinical decision rules, patient out-of-pocket cost information, and findings from malpractice case review.

Some (49 of 167; 29.3%) selected CT imaging for their simulated patient and were impervious to all information presented. It is possible that these clinicians believed the CT scan was the best test for the patient and were not influenced to change their ordering behavior. The majority of these clinicians were male, recorded > 10 years of clinical practice, and scored the lowest response on our use of resource and care in use of money questions.

The largest group of clinicians (98 of 167; 58.7%) modified their medical imaging order at least once for clinical vignette #1 when presented clinical decision rule, cost, and malpractice care review information. Of these clinicians about one third (36 of 98; 36.7%) modified their imaging order more than once.

An unexpected finding in our research was clinician medical image ordering in response to clinical vignette #2. The second case was similar to the first clinical vignette in that in neither case, when applied to the clinical decision rule, would a CT image be indicated for the patient. It appears that when presented with a new case the clinicians reverted to their original medical image ordering behavior. This may be due to the limitations of a simulation-based study or possibly due to their interpretation of vignette #2 differently than anticipated. Our study was not designed or powered to address why clinicians did not change their ordering behavior in response to clinical vignette #2.

## Conclusion

Our research contributes to the body of evidence on clinician decision making and key information that may influence medical image ordering behavior. The findings suggest that clinicians may respond to key information if presented within the context of their clinical workflow. In our study, we provided links to source documents and evidence supporting the information. Clinicians are typically decisive in their decision making with their patients. Medical informaticians and health information technologists should be extremely thoughtful in their information presentation when designing clinical decision support systems and other tools.

## Additional file

Additional file 1: Intervention slides, copy of the intervention slides that were embedded into our simulation research study. (PPTX 66 kb)

## Abbreviations

AMA: American Medical Association
ANOVA: Analysis of variance
CCHR: Canadian CT Head Rule
CME: Continuing medical education
CT: Computed tomography
ED: Emergency department
EM: Emergency medicine
