# Supplementary material for: Effect of clinical decision rules, patient cost and malpractice information on clinician brain CT image ordering: a randomized controlled trial
Source: BMC Med Inform Decis Mak. 2018 Mar 12;18:20. doi: 10.1186/s12911-018-0602-1 (PMC5848437; doi:10.1186/s12911-018-0602-1)
Supplement: Supplementary file 1 — Intervention slides, copy of the intervention slides that were embedded into our simulation research study. (PPTX 66 kb) [file 12911_2018_602_MOESM1_ESM.pptx]

## Slide 1
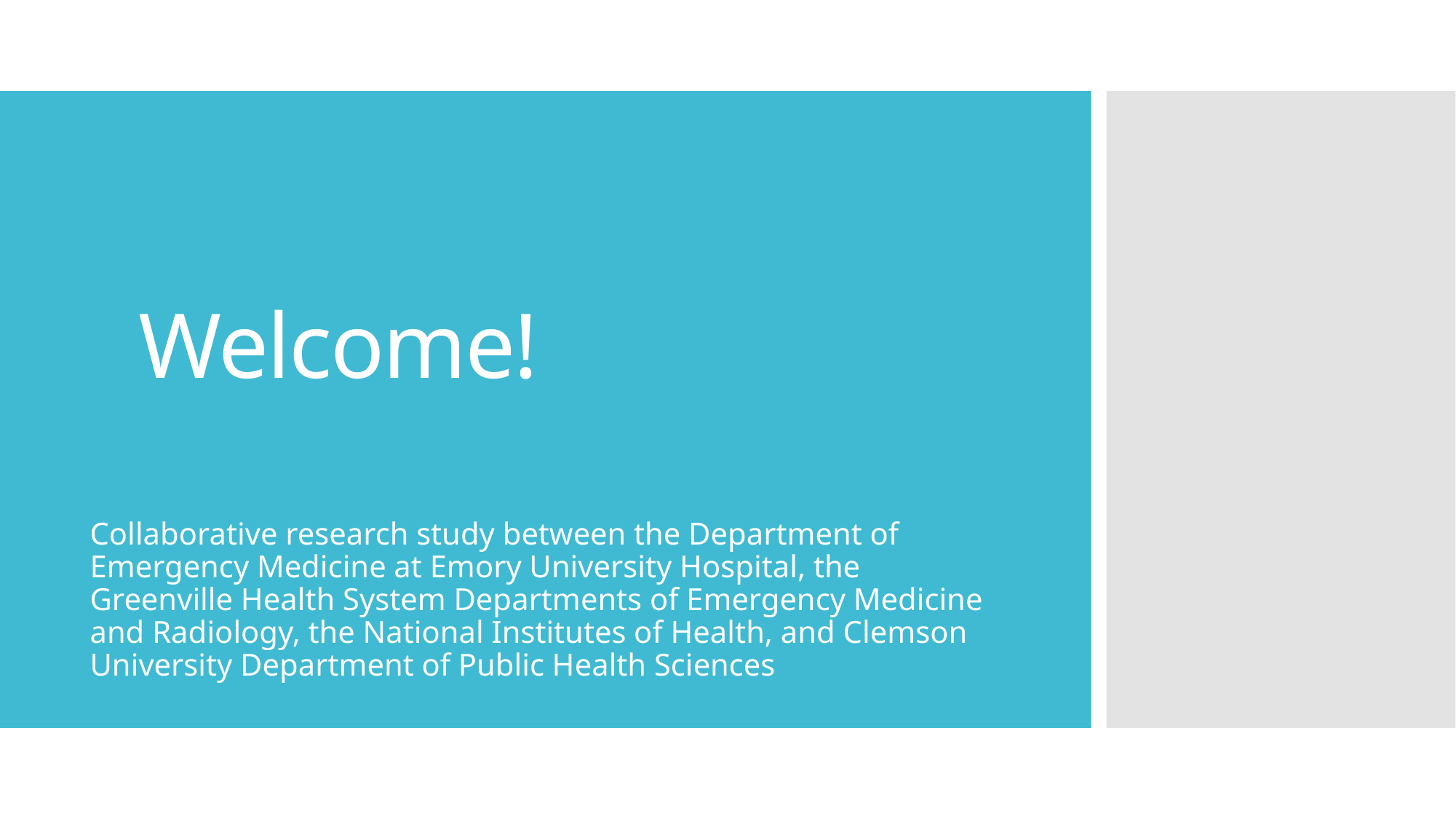

# Welcome!
Collaborative research study between the Department of Emergency Medicine at Emory University Hospital, the Greenville Health System Departments of Emergency Medicine and Radiology, the National Institutes of Health, and Clemson University Department of Public Health Sciences

## Slide 2
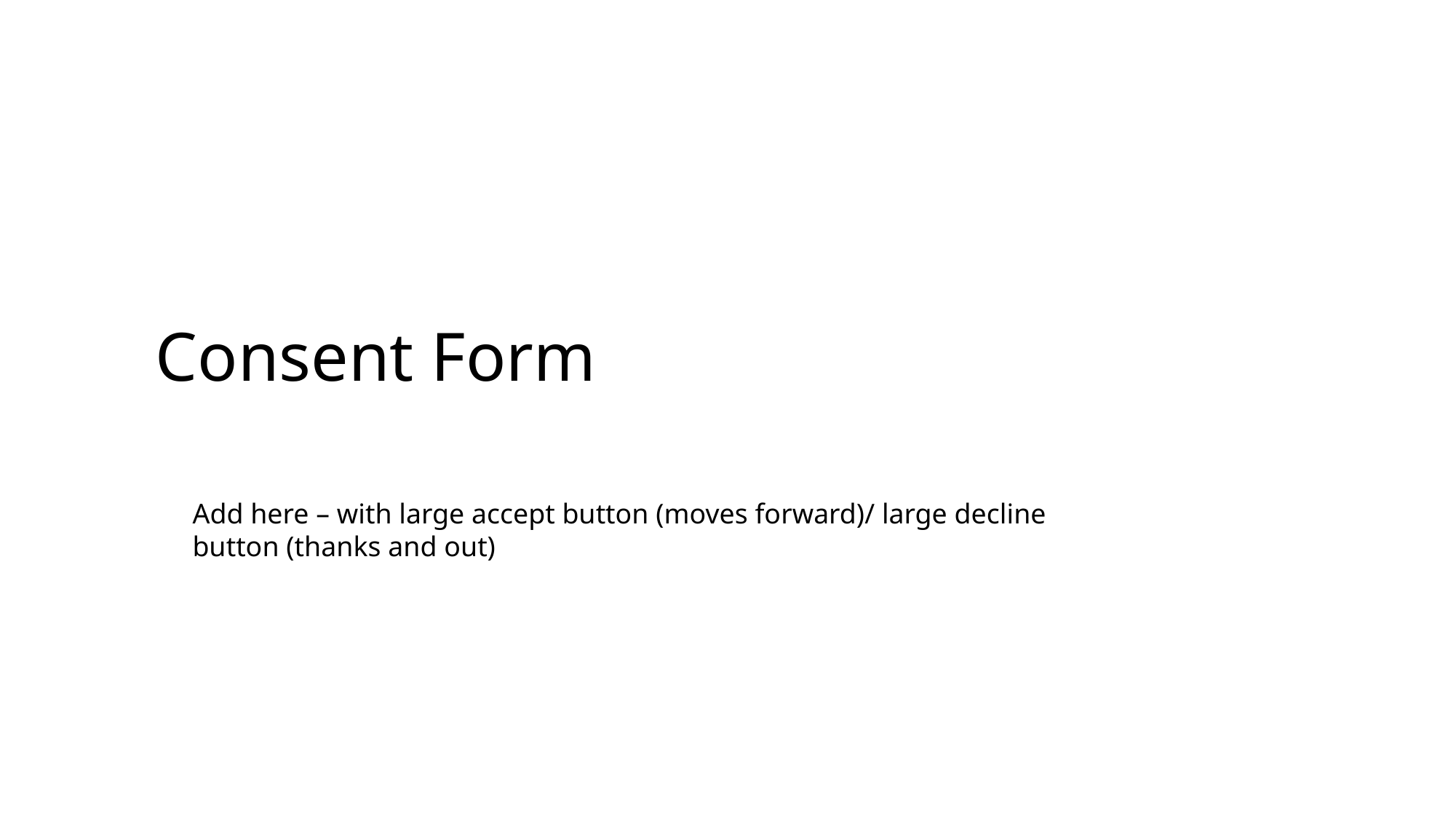

Consent Form
Add here – with large accept button (moves forward)/ large decline button (thanks and out)

## Slide 3
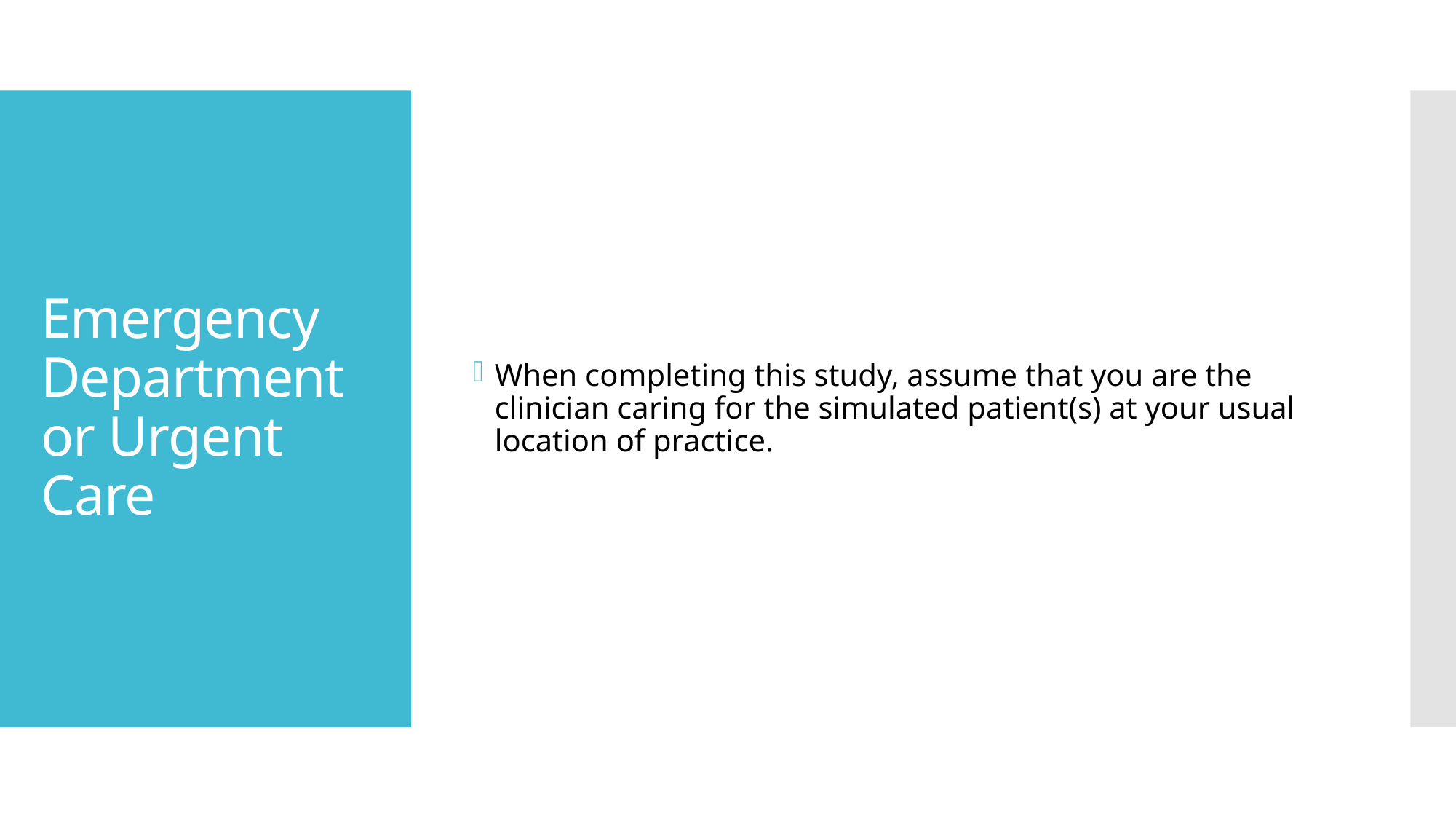

When completing this study, assume that you are the clinician caring for the simulated patient(s) at your usual location of practice.
# Emergency Department or Urgent Care

## Slide 4
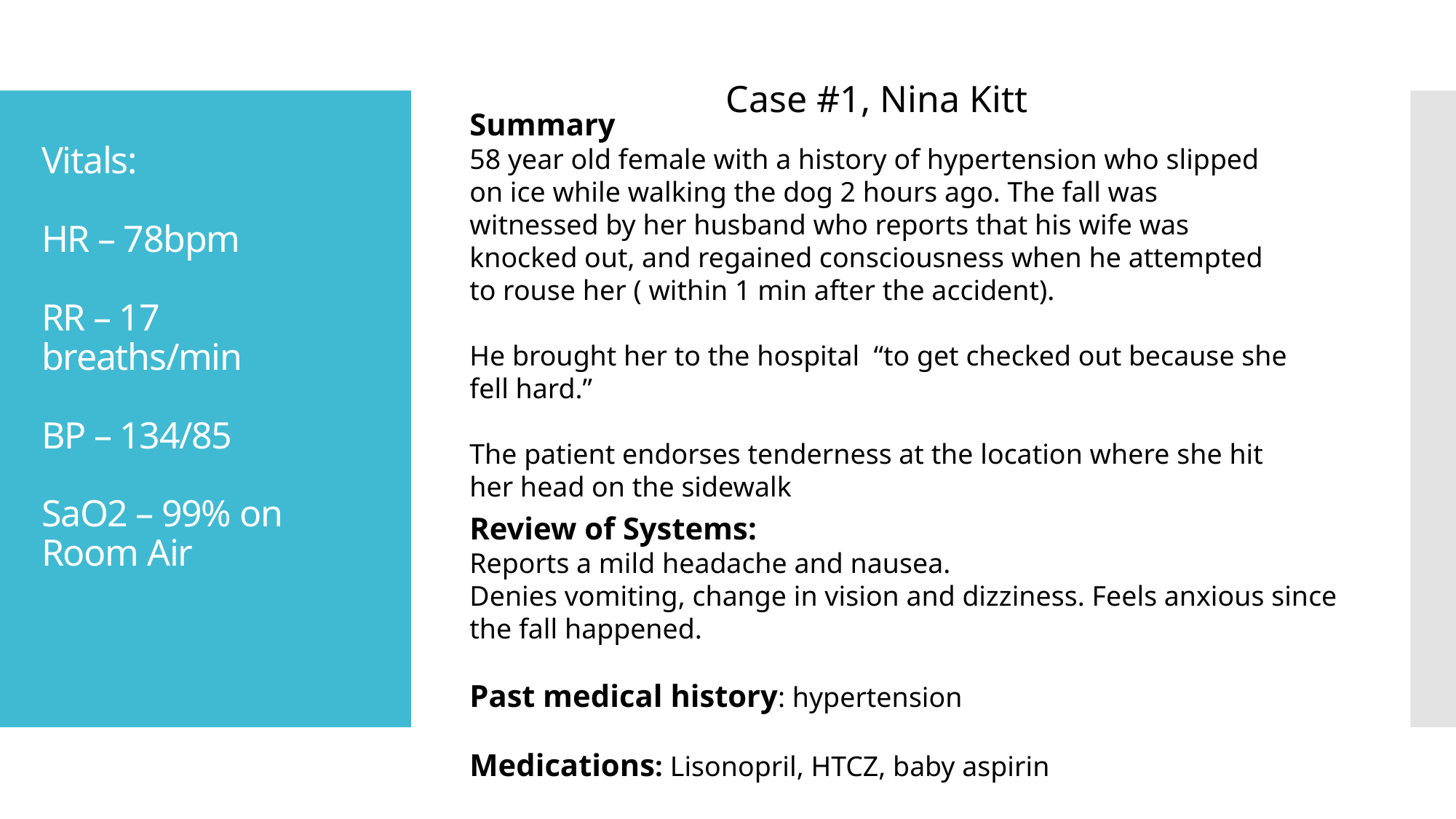

Case #1, Nina Kitt
Summary
58 year old female with a history of hypertension who slipped on ice while walking the dog 2 hours ago. The fall was witnessed by her husband who reports that his wife was knocked out, and regained consciousness when he attempted to rouse her ( within 1 min after the accident).
He brought her to the hospital “to get checked out because she fell hard.”
The patient endorses tenderness at the location where she hit her head on the sidewalk
# Vitals: HR – 78bpm RR – 17 breaths/min BP – 134/85 SaO2 – 99% on Room Air
Review of Systems:
Reports a mild headache and nausea.
Denies vomiting, change in vision and dizziness. Feels anxious since the fall happened.
Past medical history: hypertension
Medications: Lisonopril, HTCZ, baby aspirin

## Slide 5
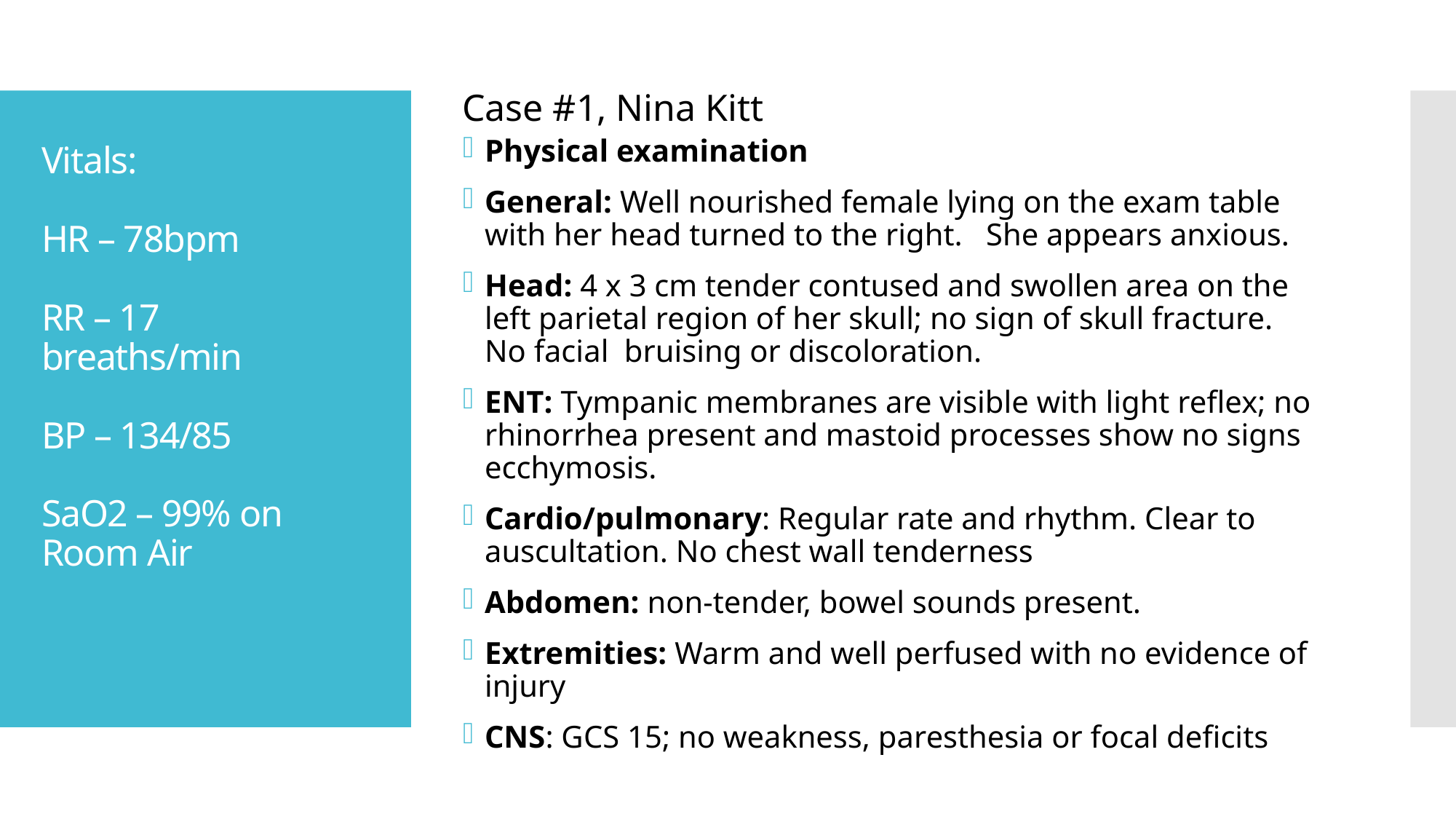

Case #1, Nina Kitt
# Vitals: HR – 78bpm RR – 17 breaths/min BP – 134/85 SaO2 – 99% on Room Air
Physical examination
General: Well nourished female lying on the exam table with her head turned to the right. She appears anxious.
Head: 4 x 3 cm tender contused and swollen area on the left parietal region of her skull; no sign of skull fracture. No facial bruising or discoloration.
ENT: Tympanic membranes are visible with light reflex; no rhinorrhea present and mastoid processes show no signs ecchymosis.
Cardio/pulmonary: Regular rate and rhythm. Clear to auscultation. No chest wall tenderness
Abdomen: non-tender, bowel sounds present.
Extremities: Warm and well perfused with no evidence of injury
CNS: GCS 15; no weakness, paresthesia or focal deficits

## Slide 6
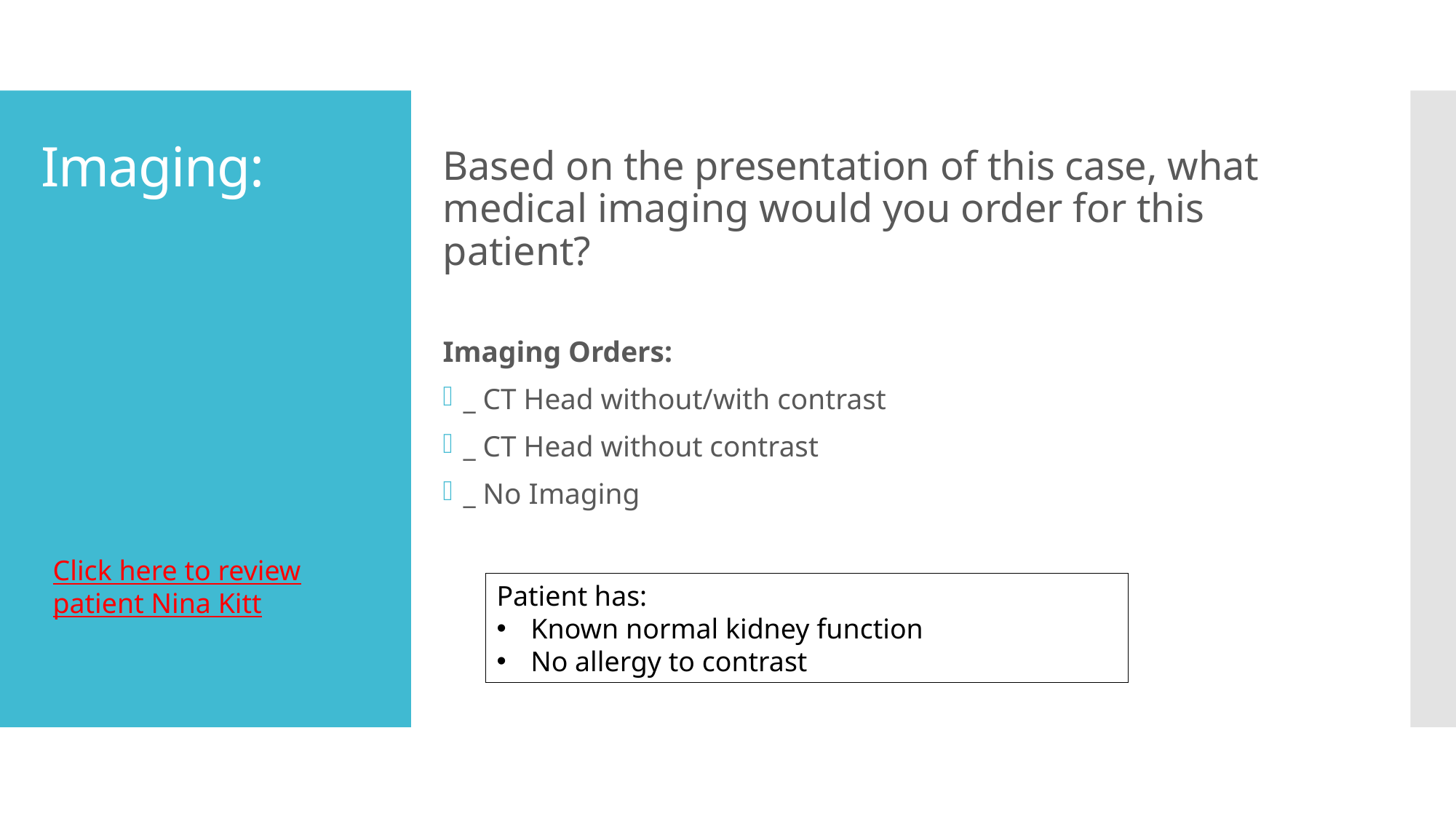

Based on the presentation of this case, what medical imaging would you order for this patient?
Imaging Orders:
_ CT Head without/with contrast
_ CT Head without contrast
_ No Imaging
# Imaging:
Click here to review patient Nina Kitt
Patient has:
Known normal kidney function
No allergy to contrast

## Slide 7
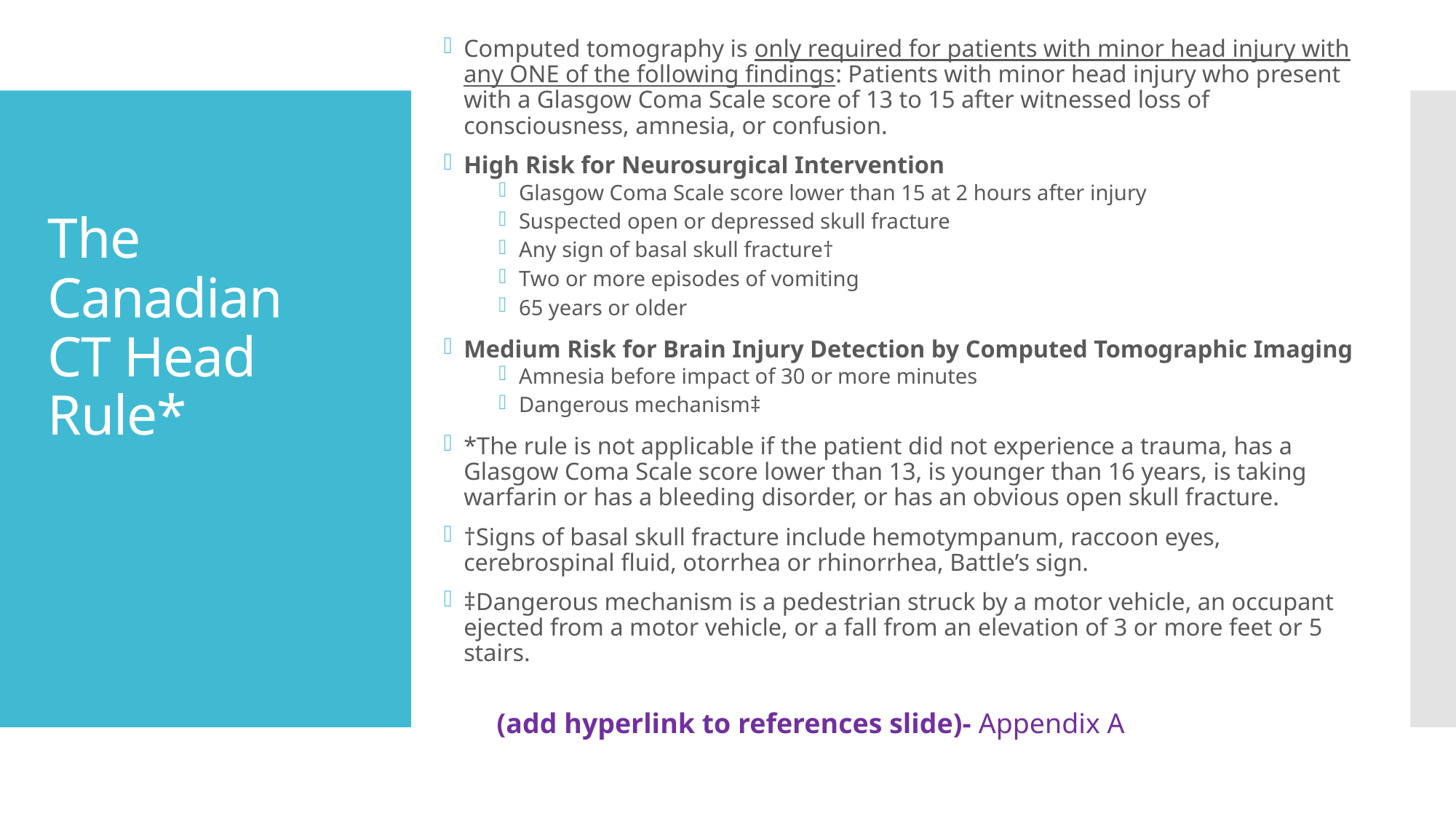

Computed tomography is only required for patients with minor head injury with any ONE of the following findings: Patients with minor head injury who present with a Glasgow Coma Scale score of 13 to 15 after witnessed loss of consciousness, amnesia, or confusion.
High Risk for Neurosurgical Intervention
Glasgow Coma Scale score lower than 15 at 2 hours after injury
Suspected open or depressed skull fracture
Any sign of basal skull fracture†
Two or more episodes of vomiting
65 years or older
Medium Risk for Brain Injury Detection by Computed Tomographic Imaging
Amnesia before impact of 30 or more minutes
Dangerous mechanism‡
*The rule is not applicable if the patient did not experience a trauma, has a Glasgow Coma Scale score lower than 13, is younger than 16 years, is taking warfarin or has a bleeding disorder, or has an obvious open skull fracture.
†Signs of basal skull fracture include hemotympanum, raccoon eyes, cerebrospinal fluid, otorrhea or rhinorrhea, Battle’s sign.
‡Dangerous mechanism is a pedestrian struck by a motor vehicle, an occupant ejected from a motor vehicle, or a fall from an elevation of 3 or more feet or 5 stairs.
# The Canadian CT Head Rule*
(add hyperlink to references slide)- Appendix A

## Slide 8
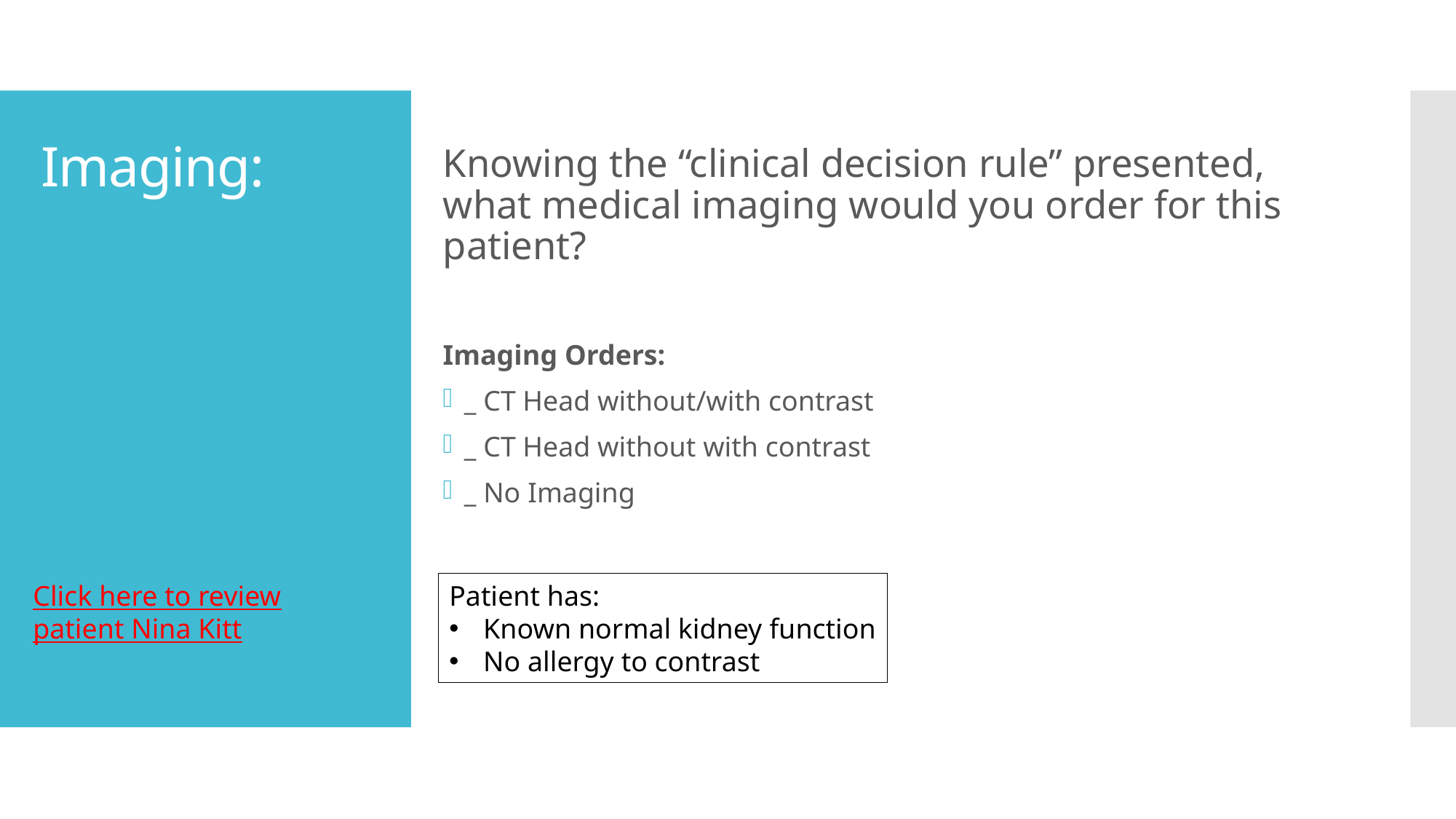

Knowing the “clinical decision rule” presented, what medical imaging would you order for this patient?
Imaging Orders:
_ CT Head without/with contrast
_ CT Head without with contrast
_ No Imaging
# Imaging:
Click here to review patient Nina Kitt
Patient has:
Known normal kidney function
No allergy to contrast

## Slide 9
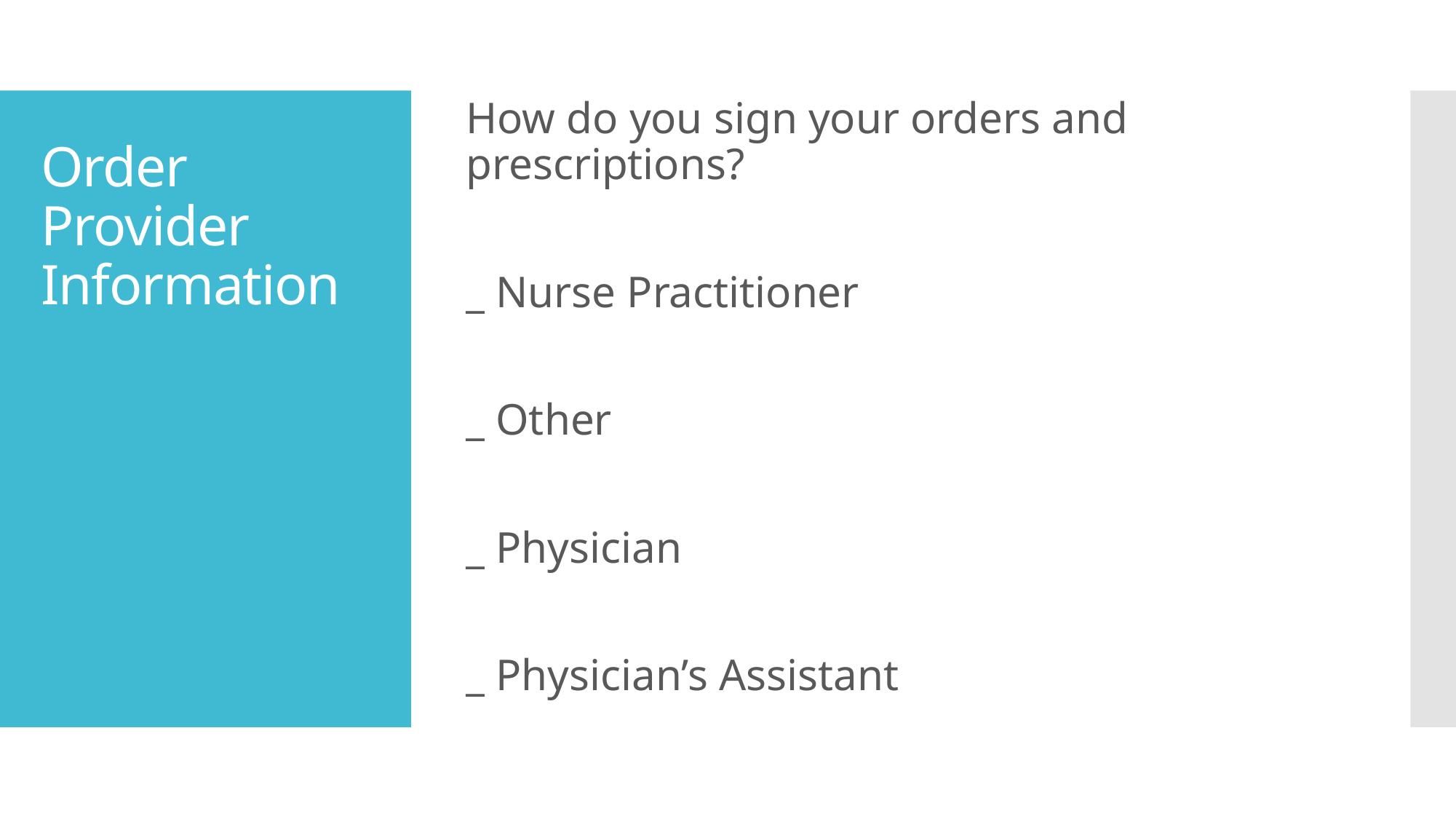

How do you sign your orders and prescriptions?
_ Nurse Practitioner
_ Other
_ Physician
_ Physician’s Assistant
# Order Provider Information

## Slide 10
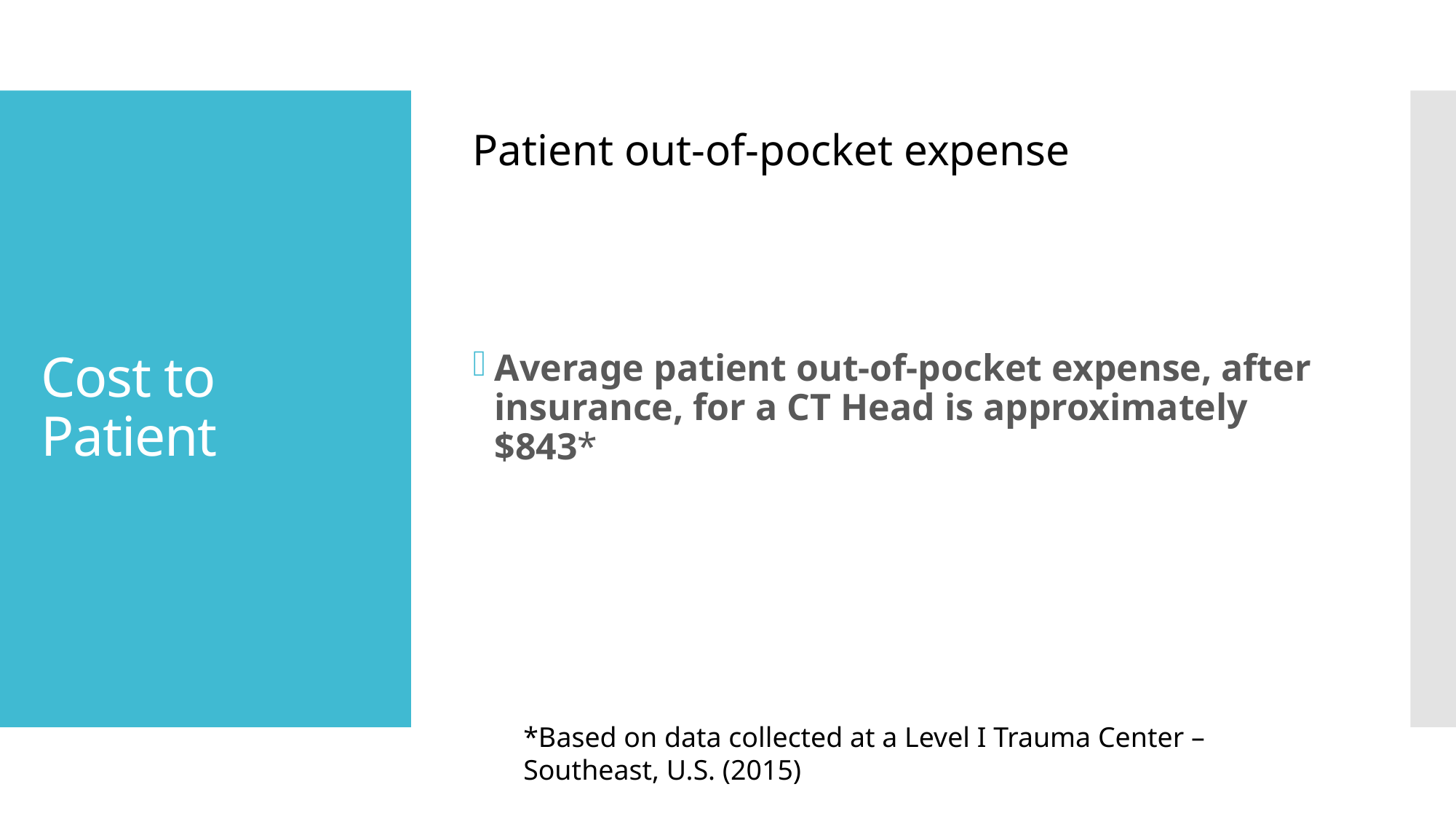

Average patient out-of-pocket expense, after insurance, for a CT Head is approximately $843*
Patient out-of-pocket expense
# Cost to Patient
*Based on data collected at a Level I Trauma Center – Southeast, U.S. (2015)

## Slide 11
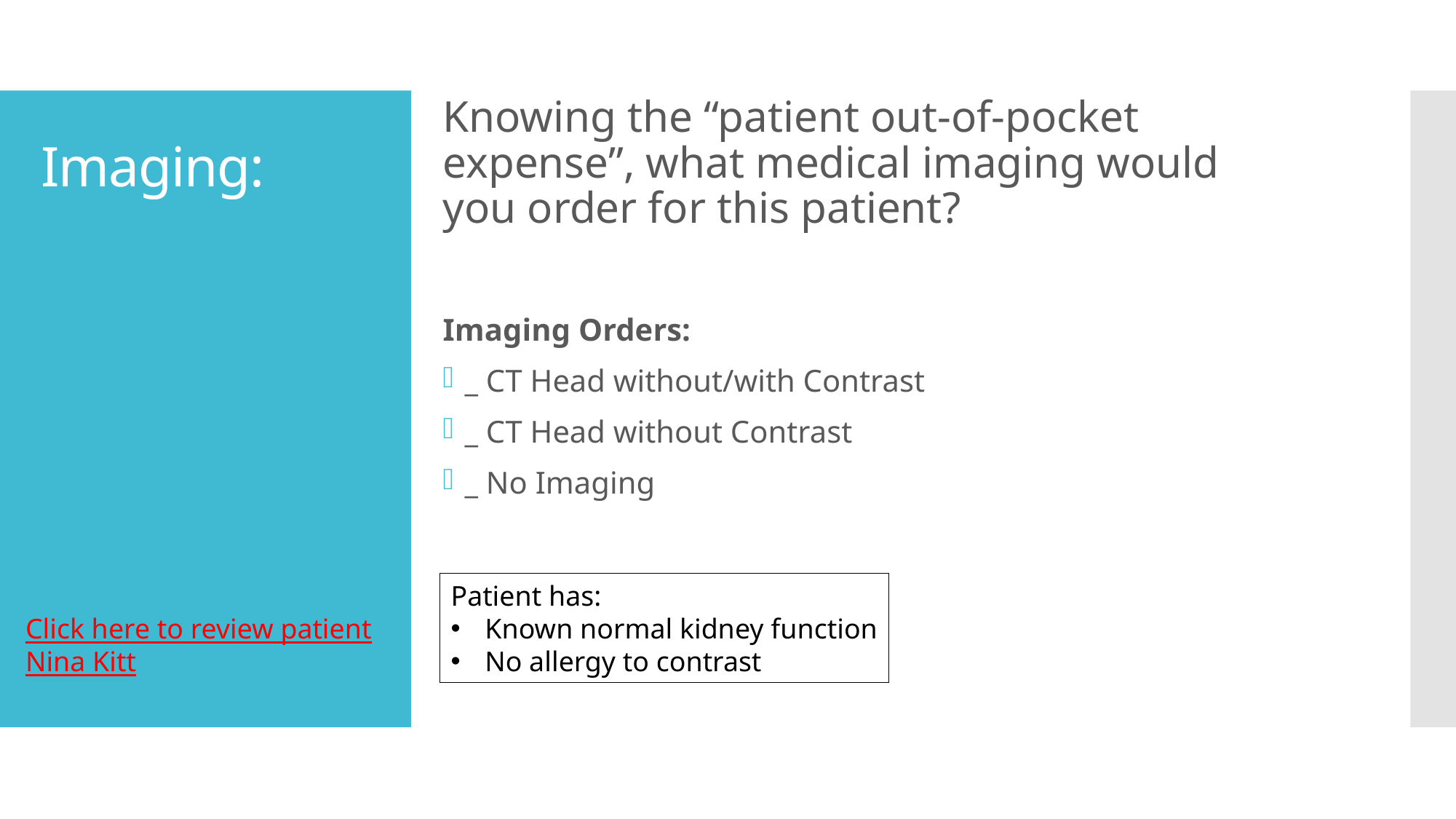

Knowing the “patient out-of-pocket expense”, what medical imaging would you order for this patient?
Imaging Orders:
_ CT Head without/with Contrast
_ CT Head without Contrast
_ No Imaging
# Imaging:
Patient has:
Known normal kidney function
No allergy to contrast
Click here to review patient Nina Kitt

## Slide 12
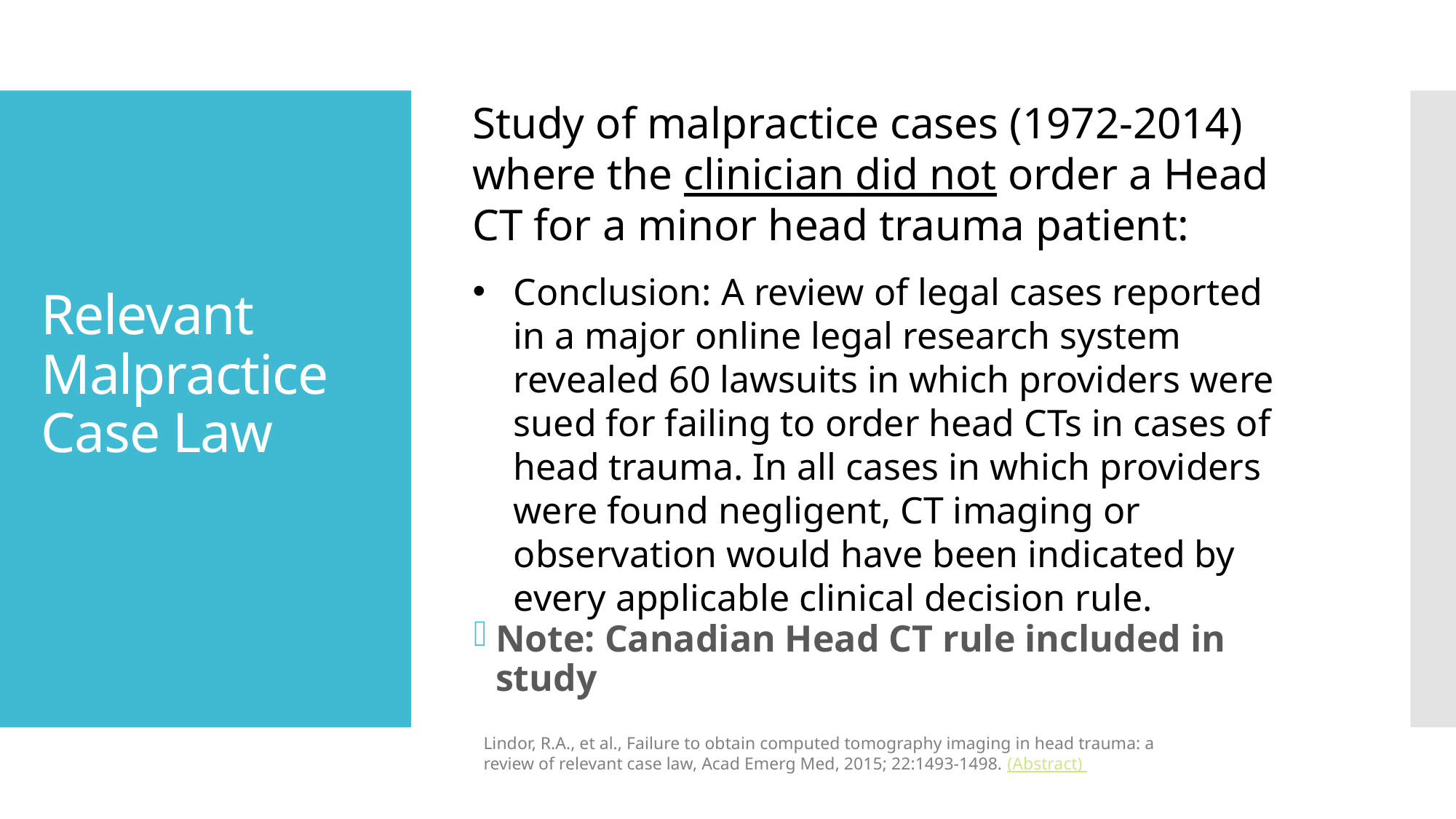

Study of malpractice cases (1972-2014) where the clinician did not order a Head CT for a minor head trauma patient:
# Relevant Malpractice Case Law
Note: Canadian Head CT rule included in study
Conclusion: A review of legal cases reported in a major online legal research system revealed 60 lawsuits in which providers were sued for failing to order head CTs in cases of head trauma. In all cases in which providers were found negligent, CT imaging or observation would have been indicated by every applicable clinical decision rule.
Lindor, R.A., et al., Failure to obtain computed tomography imaging in head trauma: a review of relevant case law, Acad Emerg Med, 2015; 22:1493-1498. (Abstract)

## Slide 13
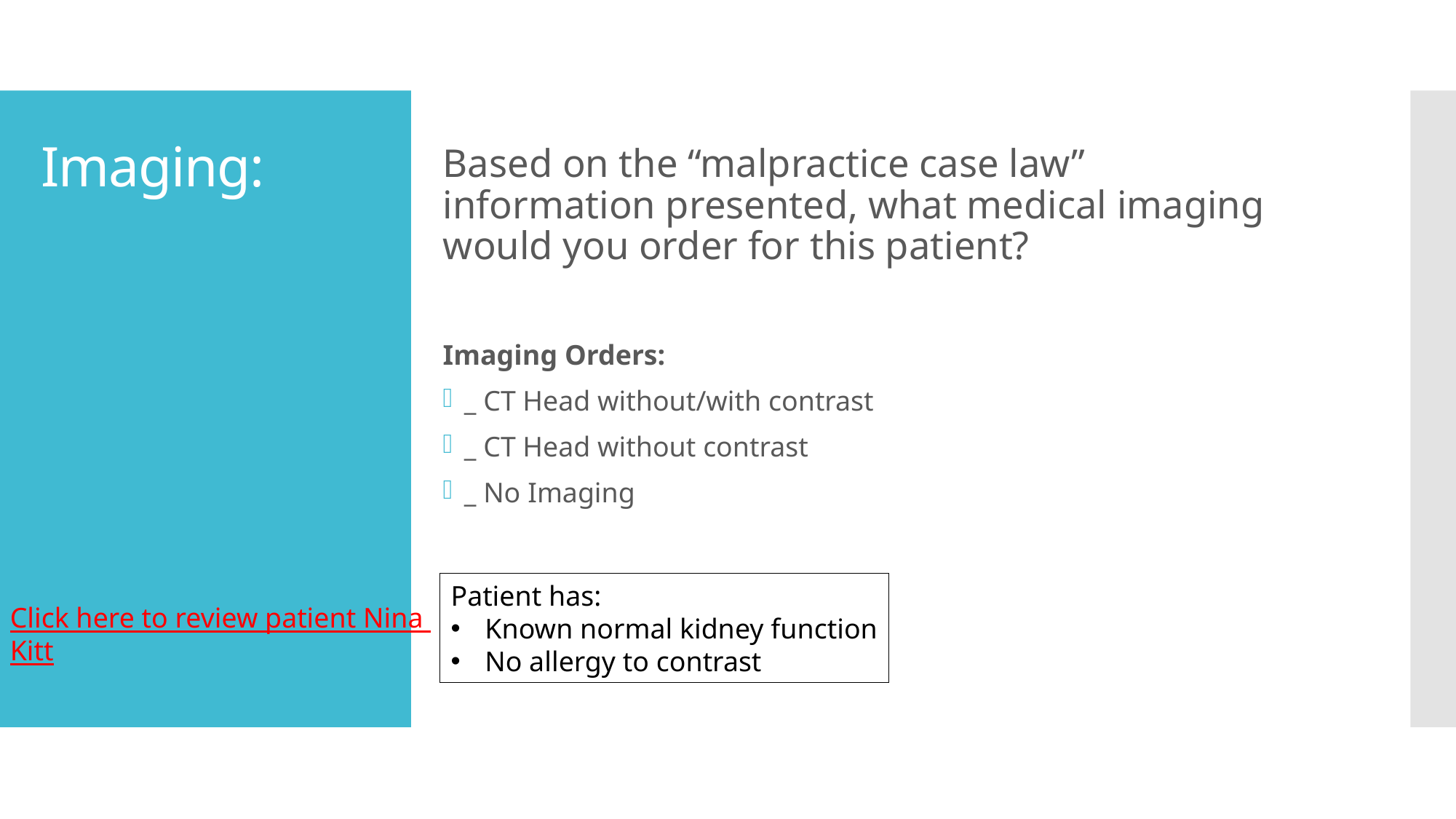

Based on the “malpractice case law” information presented, what medical imaging would you order for this patient?
Imaging Orders:
_ CT Head without/with contrast
_ CT Head without contrast
_ No Imaging
# Imaging:
Patient has:
Known normal kidney function
No allergy to contrast
Click here to review patient Nina
Kitt

## Slide 14
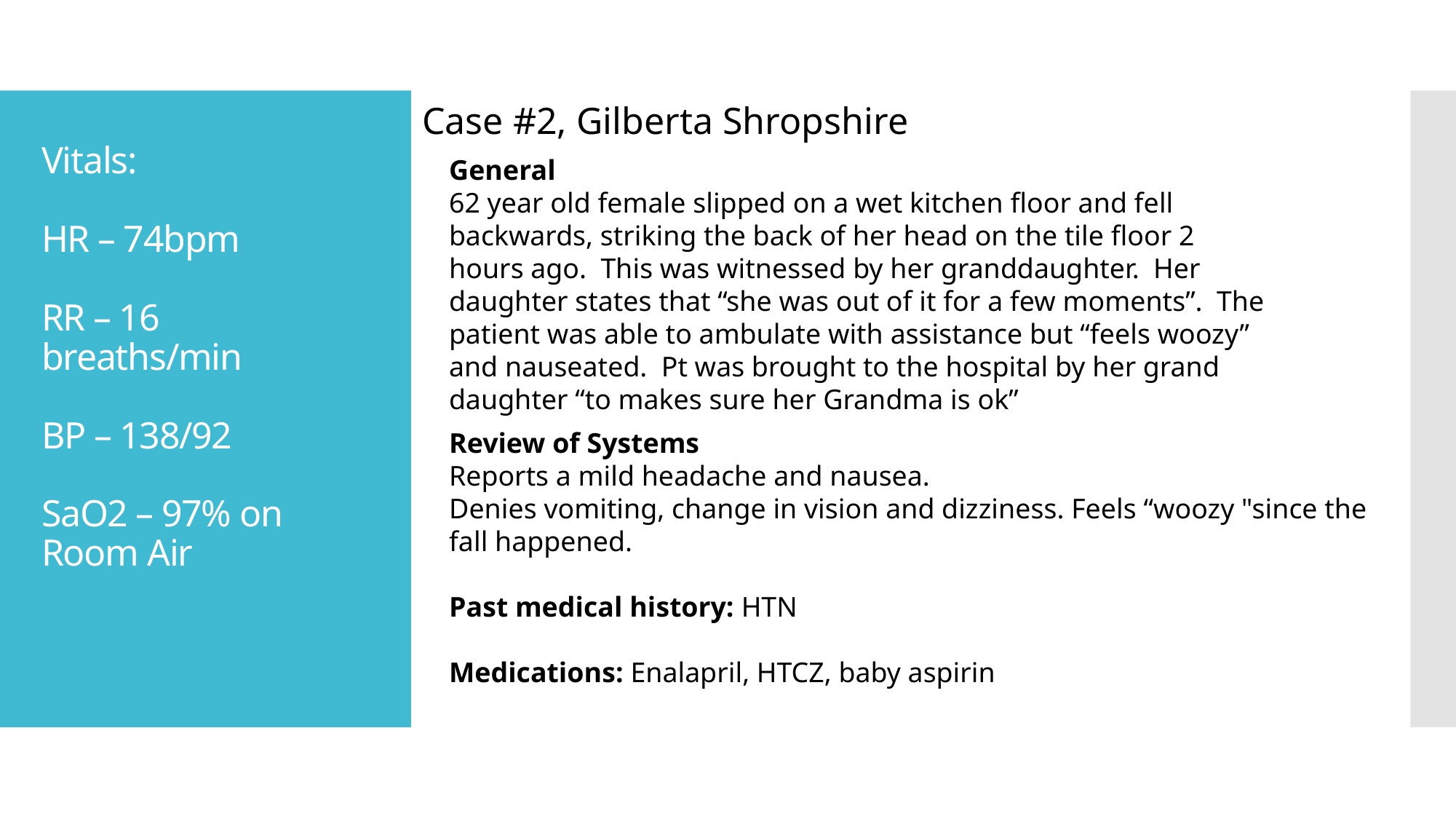

Case #2, Gilberta Shropshire
# Vitals: HR – 74bpm RR – 16 breaths/min BP – 138/92 SaO2 – 97% on Room Air
General
62 year old female slipped on a wet kitchen floor and fell backwards, striking the back of her head on the tile floor 2 hours ago. This was witnessed by her granddaughter. Her daughter states that “she was out of it for a few moments”. The patient was able to ambulate with assistance but “feels woozy” and nauseated. Pt was brought to the hospital by her grand daughter “to makes sure her Grandma is ok”
Review of Systems
Reports a mild headache and nausea.
Denies vomiting, change in vision and dizziness. Feels “woozy "since the fall happened.
Past medical history: HTN
Medications: Enalapril, HTCZ, baby aspirin

## Slide 15
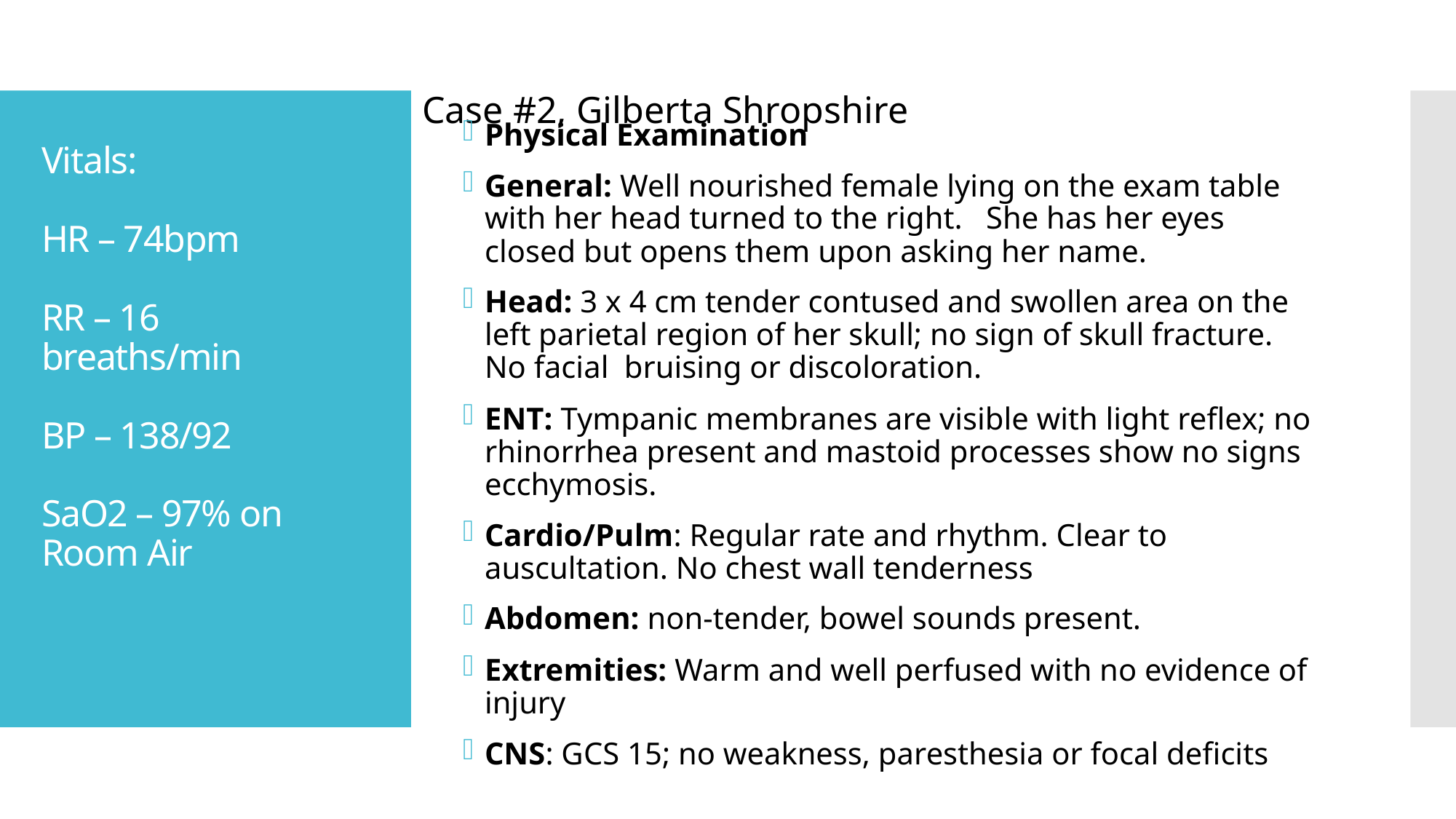

Case #2, Gilberta Shropshire
Physical Examination
General: Well nourished female lying on the exam table with her head turned to the right. She has her eyes closed but opens them upon asking her name.
Head: 3 x 4 cm tender contused and swollen area on the left parietal region of her skull; no sign of skull fracture. No facial bruising or discoloration.
ENT: Tympanic membranes are visible with light reflex; no rhinorrhea present and mastoid processes show no signs ecchymosis.
Cardio/Pulm: Regular rate and rhythm. Clear to auscultation. No chest wall tenderness
Abdomen: non-tender, bowel sounds present.
Extremities: Warm and well perfused with no evidence of injury
CNS: GCS 15; no weakness, paresthesia or focal deficits
# Vitals: HR – 74bpm RR – 16 breaths/min BP – 138/92 SaO2 – 97% on Room Air

## Slide 16
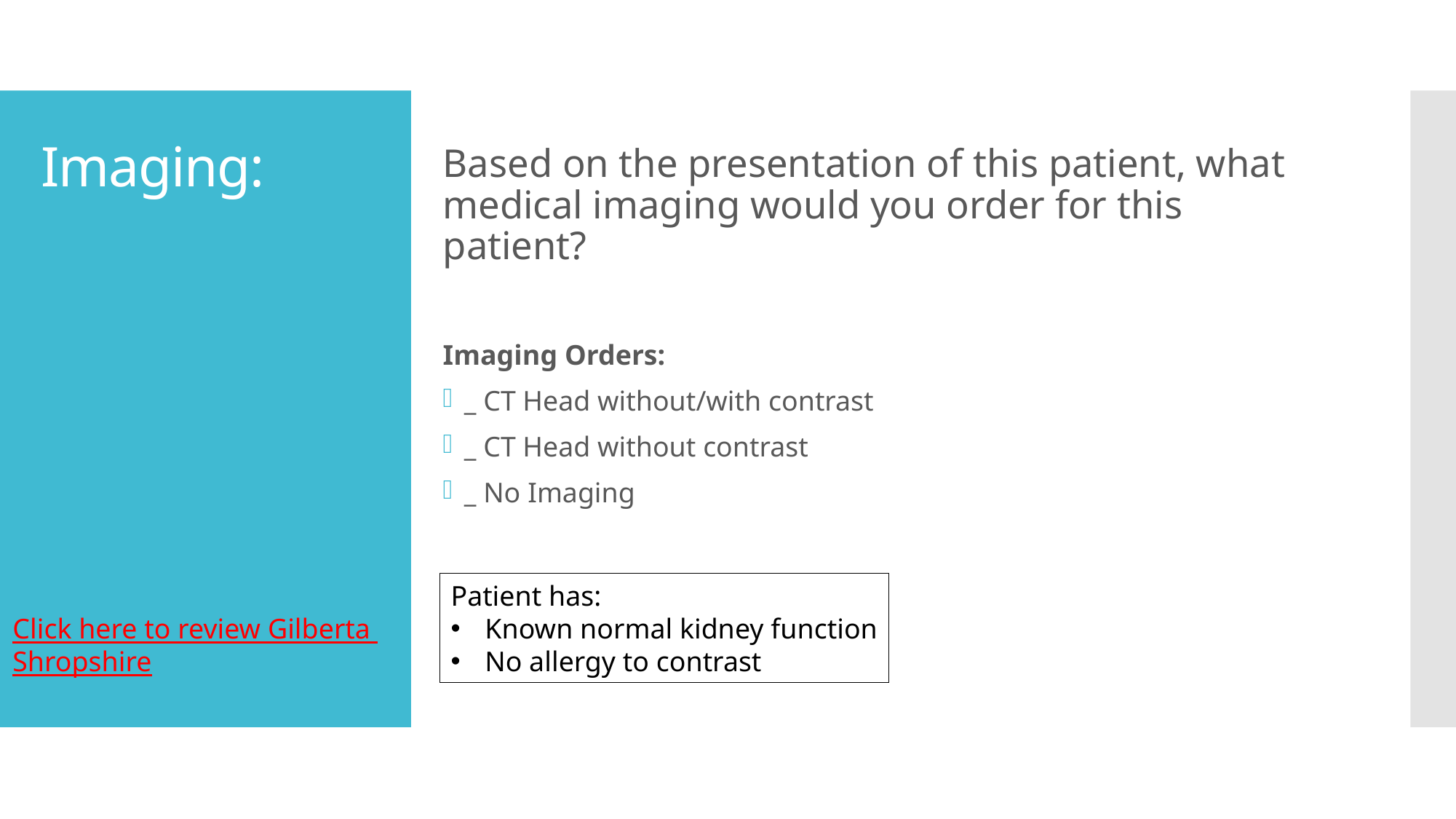

Based on the presentation of this patient, what medical imaging would you order for this patient?
Imaging Orders:
_ CT Head without/with contrast
_ CT Head without contrast
_ No Imaging
# Imaging:
Patient has:
Known normal kidney function
No allergy to contrast
Click here to review Gilberta
Shropshire

## Slide 17
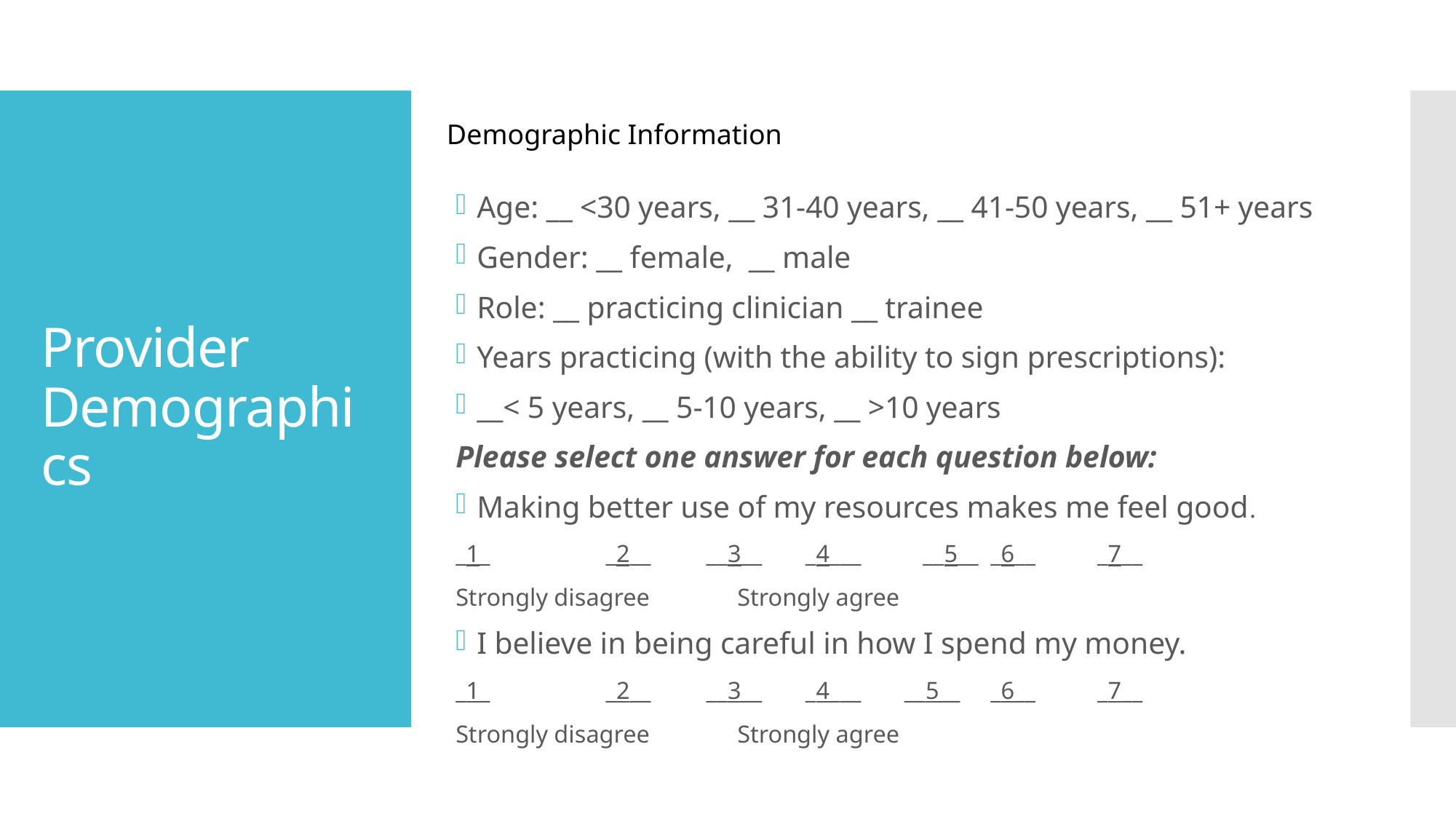

Demographic Information
# Provider Demographics
Age: __ <30 years, __ 31-40 years, __ 41-50 years, __ 51+ years
Gender: __ female, __ male
Role: __ practicing clinician __ trainee
Years practicing (with the ability to sign prescriptions):
__< 5 years, __ 5-10 years, __ >10 years
Please select one answer for each question below:
Making better use of my resources makes me feel good.
_1_	 _2__ __3__ _4___ __5__	_6__	_7__
Strongly disagree					 Strongly agree
I believe in being careful in how I spend my money.
_1_	 _2__ __3__ _4___ __5__	_6__	_7__
Strongly disagree					 Strongly agree

## Slide 18
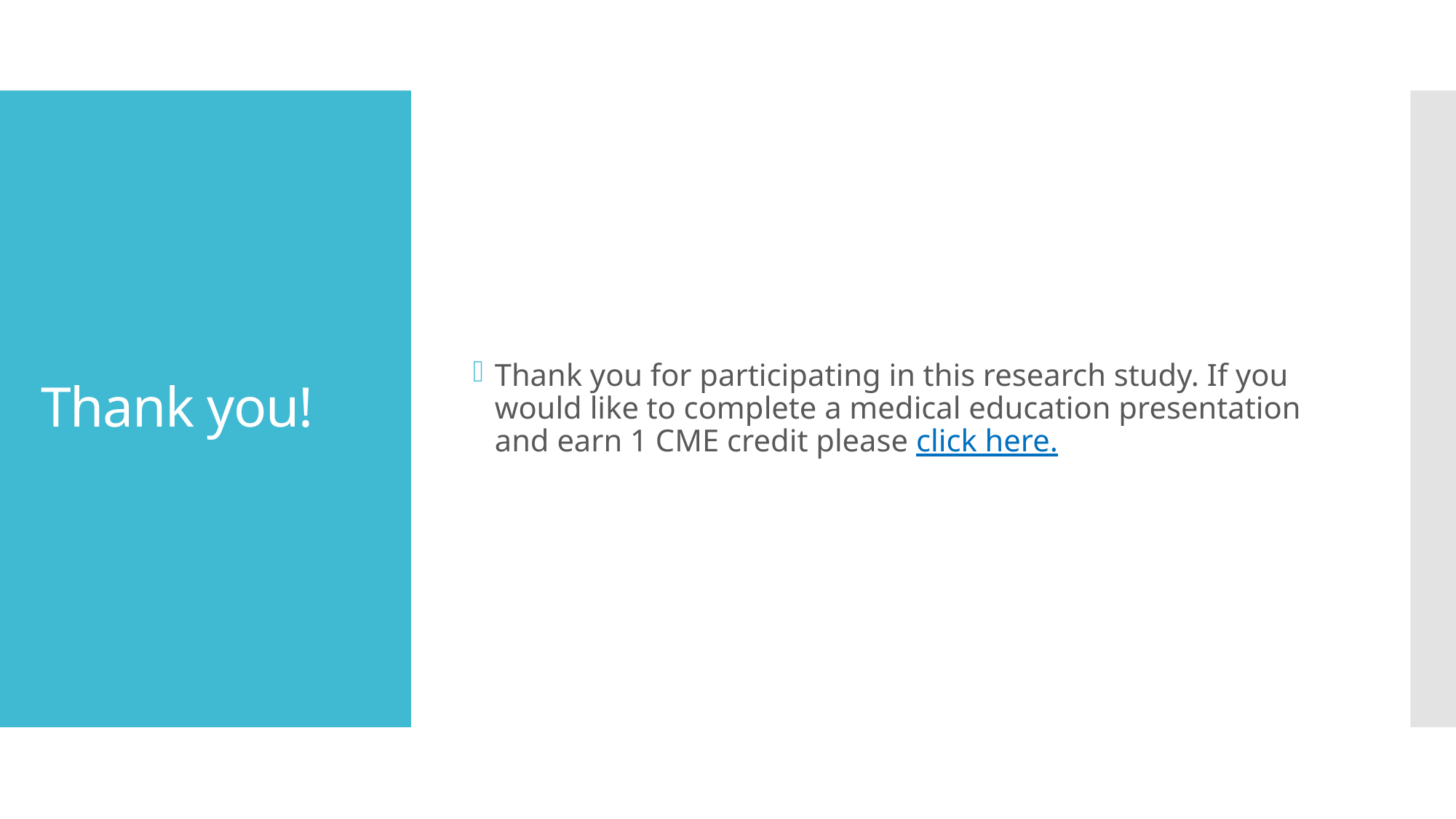

Thank you for participating in this research study. If you would like to complete a medical education presentation and earn 1 CME credit please click here.
# Thank you!
